# Nrf2-Keap1 pathway promotes cell proliferation and diminishes ferroptosis

**DOI:** 10.1038/oncsis.2017.65

**Published:** 2017-08-14

**Authors:** Z Fan, A-K Wirth, D Chen, C J Wruck, M Rauh, M Buchfelder, N Savaskan

**Affiliations:** 1 Translational Cell Biology and Neurooncology Laboratory at the Department of Neurosurgery, University Medical School Hospital Universitätsklinikum Erlangen (UKER), Friedrich-Alexander University of Erlangen–Nürnberg (FAU), Erlangen, Germany; 2 Laboratory of Exercise and Health, Institute of Movement Sciences, Department of Health Sciences and Technology, (D-HEST), ETH Zürich, Schwerzenbach, Switzerland; 3 Department of Gene Vectors, Helmholtz Zentrum München, German Research Center for Environmental Health, Munich, Germany; 4 Department of Otolaryngology–Head and Neck Surgery, Chinese PLA General Hospital, Beijing, China; 5 Institute of Anatomy and Cell Biology, Universitätsklinikum RWTH Aachen, Aachen, Germany; 6 Department of Pediatrics and Adolescent Medicine, University Medical School Hospital Erlangen (UKER), Friedrich-Alexander University of Erlangen–Nürnberg (FAU), Erlangen, Germany; 7 BiMECON, Berlin, Germany

## Abstract

Cancer cells are hallmarked by high proliferation and imbalanced redox consumption and signaling. Various oncogenic pathways such as proliferation and evading cell death converge on redox-dependent signaling processes. Nrf2 is a key regulator in these redox-dependent events and operates in cytoprotection, drug metabolism and malignant progression in cancer cells. Here, we show that patients with primary malignant brain tumors (glioblastomas, WHO °IV gliomas, GBM) have a devastating outcome and overall reduced survival when Nrf2 levels are upregulated. Nrf2 overexpression or Keap1 knockdown in glioma cells accelerate proliferation and oncogenic transformation. Further, activation of the Nrf2-Keap1 signaling upregulates xCT (aka SLC7A11 or system X_c_^−^) and amplifies glutamate secretion thereby impacting on the tumor microenvironment. Moreover, both fostered Nrf2 expression and conversely Keap1 inhibition promote resistance to ferroptosis. Altogether, the Nrf2-Keap1 pathway operates as a switch for malignancy in gliomas promoting cell proliferation and resistance to cell death processes such as ferroptosis. Our data demonstrate that the Nrf2-Keap1 pathway is critical for cancer cell growth and operates on xCT. Nrf2 presents the Achilles’ heel of cancer cells and thus provides a valid therapeutic target for sensitizing cancer for chemotherapeutics.

## Introduction

A common concept concerning the development of cancer considers the specific deregulation of genes associated with the redox system.^1, 2^ As the transcription of most genes is regulated by redox-sensitive transcription factors, a mutation or deregulation of transcription factors might have an even bigger biological impact on the regulation of redox homeostasis and cell metabolism. One important transcription factor in this concert represents the nuclear factor (erythoid-derived)-like 2 (Nrf2). Nrf2 was first isolated and characterized by Moi *et al.*^3^ Nrf2 is a member of the cap'n'collar subfamily of transcription factors and contains a basic leucine zipper DNA-binding domain at the C-terminus.^4^ The transcription factor is widely expressed in all tissues at low basal levels,^3^ yet not essential for normal development of mice.^5^ Under normoxic cellular conditions, Nrf2 is bound by Kelch-like ECH-associated protein 1 (Keap1) and persists in an inactivated status through ubiquitination and degradation in the proteasome.^6^

In the case when cells are exposed to increased oxidative stress, electrophiles or cytotoxic agents, Nrf2 becomes unleashed from the Keap1 binding and translocate to the nucleus. There, Nrf2 transcripts antioxidant responsive element (ARE) -dependent genes in order to balance oxidative mediators and maintain cellular redox homeostasis.^7^ In contrast, Keap1 operates as a molecular switch for activation of Nrf2 since Keap1 is able to sense and transmit oxidative challenges.^4^ Thereby, Keap1 can turn the Nrf2-mediated response on- and off-dependent on the intracellular redox status.^4^

Nrf2 has many target genes such as intracellular redox-balancing proteins like glutamate-cysteine ligase, heme oxygenase-1 (HEM-1) and glutathione peroxidases (GPX), phase II detoxifying enzymes like glutathione-S-transferase (GST), NAD(P)H quinone oxidoreductase-1 (NQO1) and multidrug resistance-associated proteins^4, 8^ These downstream effectors have a crucial role in cellular defense mechanisms. Thus, Nrf2 represents a key switch for the cellular adaptation and survival under oxidative challenges.

Nevertheless, Nrf2 could be seen as a double-edged sword since imbalanced Nrf2 levels have been reported to be involved in the development and progression of various cancers including hepatocellular carcinomas.^9^ Moreover, loss-of-function of Keap1, either by mutation or by decreased expression due to hyper-methylation of the Keap1 promoter as well as mutations in the genomic structure coding for Nrf2, has also been shown to have a role in the development of different types of cancer, for example, breast cancer and lung adenocarcinoma.^4^ These findings indicate a critical role of Nrf2 in normal tissue and tumor cells. Hence, consistent activation of the antioxidant system through the detection of Nrf2 and Keap1 mutations has been confirmed in various human tumors such as in skin, lung, larynx and esophagus cancer.^10, 11^

One of the key genes regulated by Nrf2 is xCT and it’s associated with cytoprotection.^12, 13, 14^ xCT (aka SLC7a11 or system X_c_^−^) together with CD98, forms a glutamate-cystine antiporter system that exchanges glutamate out of the cell and cystine into the cell with a 1:1 ratio. Intracellularly, cystine becomes reduced to cysteine which is a rate-limiting amino acid for the production of cellular protector glutathione (GSH). Moreover, xCT has a crucial role in the cellular antioxidant system, in particular in primary brain tumors.^15, 16, 17^ xCT is involved in glioma-induced neuronal cell death, perifocal edema^18^ and tumor-associated epileptic events.^19^

Further, targeting the xCT system may be a promising therapeutic strategy for the treatment of malignant gliomas. Therefore, we went on investigating Nrf2-Keap1 as the xCT regulator. There are only a few reports about the role of Nrf2-Keap1 in brain tumors. The aim of this work was to unravel the contribution of Nrf2-Keap1 to tumor growth, malignancy and ferroptosis in gliomas. We found that Nrf2 overexpression or Keap1 knockdown foster the malignancy of gliomas and accelerate progression of glioma cells. Moreover, we revealed mechanistically that Nrf2-Keap1 operates on the regulation of the cystine-glutamate antiporter xCT in gliomas and subsequently regulates their resistance to ferroptotic cell death. Thus, Nrf2 functions in gliomas as a malignancy switch and thereby impacts the extracellular composition of the tumor microenvironment.

## Results

### Nrf2 upregulation is correlated with poorer survival outcomes in patients with brain tumors

**Figure 1:**
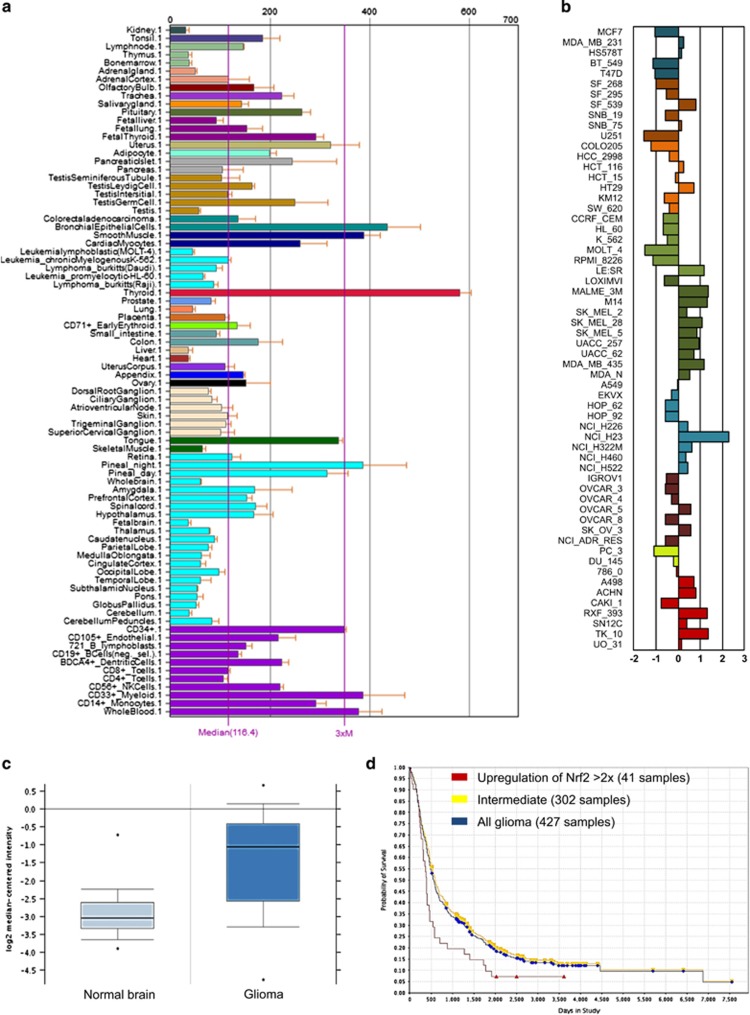
Increased Nrf2 expression in human gliomas and in glioma progression. (**a**) BioGPS database shows Nrf2 expression in different human tissues. Brain tissue shows, with exception of the pineal gland, intermediate to low expression levels compared to thyroid and epithelial cells. (**b**) CellMiner analysis of Nrf2 expression in the NCI60 tumor cell lines panel. (**c**) Comparison of 81 GBM specimens and 23 normal brain tissue samples (Oncomine) for Nrf2 mRNA expression. GBM tissues show a significant (*P*=8.67 × 10^−11^) higher expression of Nrf2. (**d**), Rembrandt database analysis of survival rates from glioma patients with different Nrf2 expression levels. Red line=upregulation of Nrf2 >2 × (41 samples), yellow=intermediate (302 samples), blue=all glioma (427 samples). Nrf2 upregulation leads to significant (*P*=0.007) poorer overall survival rates.

We first investigated the expression pattern of Nrf2 in different human organs and cell lines. Analysis of Nrf2 expression in publicly available open access databases such as BioGPS database revealed its intermediate to low expression in neuronal tissues compared with other tissues (Figure 1a). Further investigations of the NCI60 cancer cell panel covering the most established cell lines in the cancer research community revealed that Nrf2 expression is elevated mostly in skin cancer cell lines, whereas solely two glioma cell lines (SF_539, SNB75) show increased Nrf2 levels (Figure 1b). We next analyzed the Nrf2 levels in human glioblastoma (GBM) samples. In comparison to non-cancerous transformed tissue, human GBMs show elevated Nrf2 levels ranging from up to threefold increased expression in comparison to normal brain tissue serving as controls (Figure 1c). Further analysis revealed that human GBM tumors with increased Nrf2 expression (at least two times elevation or more) result in poorer clinical outcome in glioblastoma patients with overall reduced survival (Figure 1d).

### Nrf2 overexpression in gliomas shows enhanced proliferation and colony forming activity

**Figure 2:**
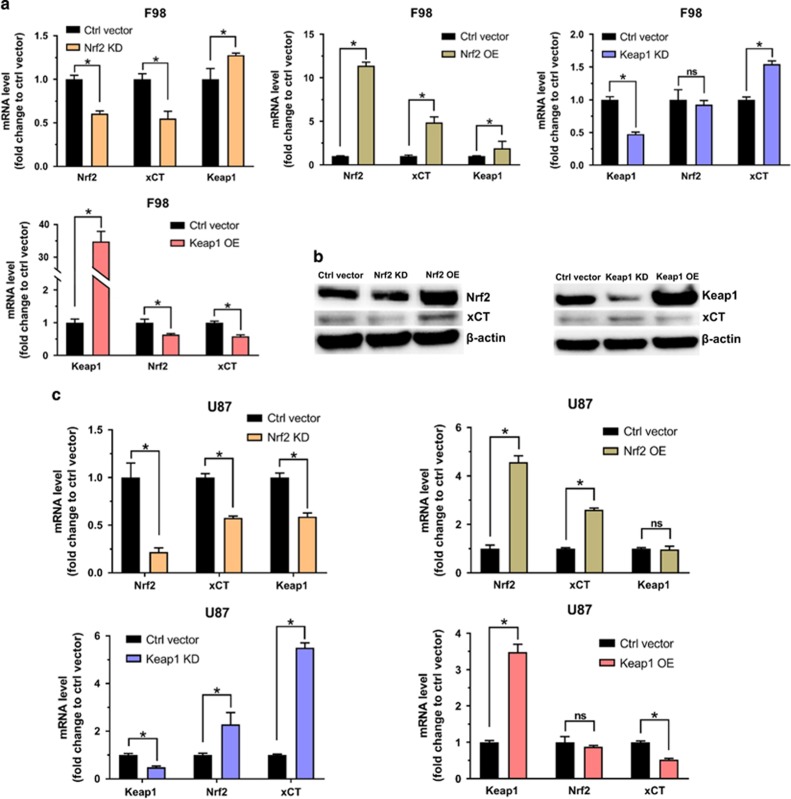
Nrf2 and Keap1 regulates xCT expression. (**a**) The impact of Nrf2/Keap1 on xCT expression in mRNA level. Quantitive real-time PCR tested the fold change of Nrf2, xCT and Keap1 mRNA level in F98 glioma cells transfected with different vectors (Nrf2 KD, Nrf2 OE, Keap1 KD, Keap OE), control vector transfected F98 glioma cells served as control and normalized to 1. Values were given as mean±s.d. Statistical significance was tested by Two-way ANOVA, *P<0.05, ns: not significant, *n*=3. (**b**) The impact of Nrf2/Keap1 on xCT protein levels. Western blot of Nrf2, Keap1 and xCT protein in F98 gliomas with different Nrf2 or Keap1 levels. Control vector transfected F98 glioma cells served as controls. β-actin was used as a loading control. (**c**) Quantitative real-time PCR analysis of Nrf2, xCT and Keap1 mRNA level in U87 glioma cells transfected with different vectors (Nrf2 KD, Nrf2 OE, Keap1 KD, Keap OE). Control vector transfected U87 glioma cells served as controls and were normalized to 1. Values were given as mean±s.d. Statistical significance was tested by two-way ANOVA, *P<0.05, ns: not significant, with *n*=3. ANOVA, analysis of variance.

To understand the biological role of Nrf2 in glioblastoma patients we next generated glioma cells with challenged Nrf2 expressions. For this we transfected rodent F98 and human U87 glioma cells with six different expressions constructs: reporter plasmid vector (Ctrl vector), Nrf2 wild-type overexpressing vector (Nrf2 OE), dominant negative Nrf2 expression vector (Nrf2 DN) and Nrf2 siRNA knockdown vector (Nrf2 KD). Moreover, we facilitated constructs for Keap1 knockdown (Keap1 KD) and Keap1 overexpression (Keap1 OE). To prove the biological function of these constructs we performed quantitative real-time PCR analyses and immunoblot analyses. Our studies revealed that Nrf2 knockdown in F98 rodent glioma cells and in human glioma U87 cells show a 40% and 78% reduced Nrf2 expression, respectively (Figures 2a and c). Nrf2 overexpression in F98 and U87 cells yielded in 11 and 4.5 times higher Nrf2 mRNA levels, respectively in comparison to control vector transfected cells (Figures 2a and c). Moreover, cells overexpressing Keap1 revealed a 35 times Keap mRNA increase in F98 cells and 3.5 times increase in U87 cells compared to control cells (Figures 2a and c). Interestingly, high expression of Keap1 significantly decreased the expression of Nrf2 by 40% in F98 cells (Figures 2a and c). However, human Keap1 OE cells showed no significant reduction in Nrf2 expression. On the other hand, cells with a low Keap1 expression through siRNA-mediated knockdown (50% in both cell lines) showed augmented Nrf2 expression (2 times higher than controls) in U87, although not in rodent F98 cells (Figures 2a and c). Furthermore, we also tested the expression levels of xCT in different groups. Nrf2 knockdown or Keap1 overexpression clearly resulted in reduced xCT mRNA (~50% decrease in both cell lines). Conversely, decreased Keap1 expression or Nrf2 overexpression significantly boosted xCT mRNA levels (up to 5 times elevation) (Figures 2a and c). Western blot analysis revealed that xCT protein levels increased in Nrf2 OE and Keap1 knockdown glioma cells (Figure 2b). Conversely, xCT protein levels decreased when Nrf2 was knocked down or when Keap1 was overexpressed (Figure 2b).

**Figure 3:**
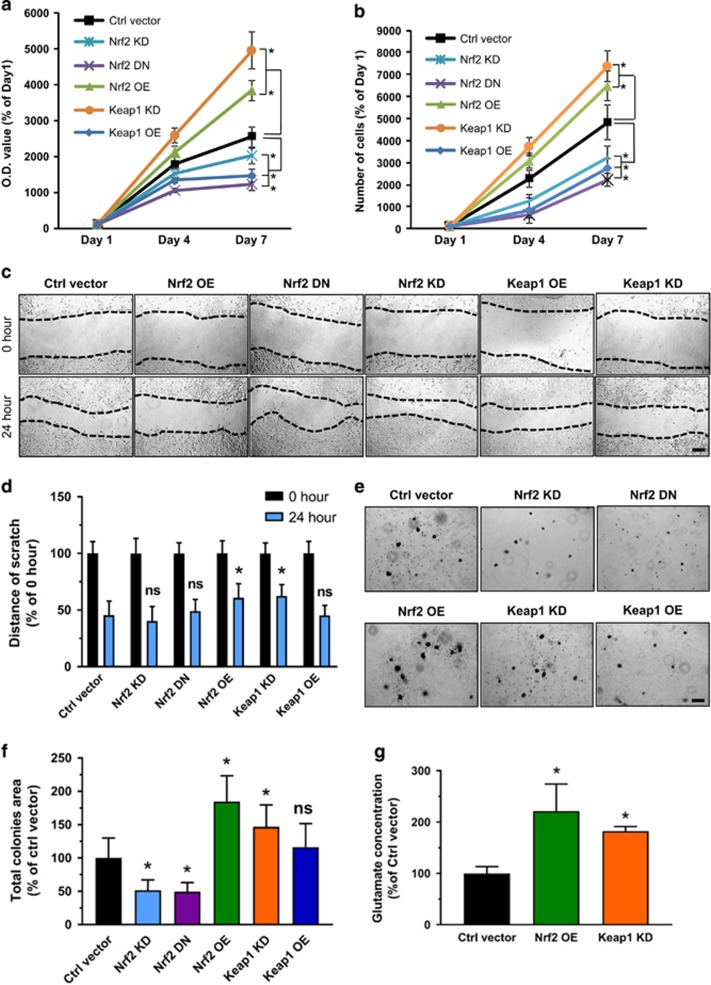
Nrf2-Keap1 pathway promotes oncogenic progression. (**a**) Proliferation analysis of rodent F98 glioma cells with different Nrf2/Keap1 expresssion. Keap1 KD and Nrf2 OE cells showed a significant higher proliferation rate compared to control cells (Ctrl vector). In contrast, proliferation is lower in Nrf2 DN, Nrf2 KD and Keap1 OE cells. Values are given as mean±s.d. Statistical significance was tested with one-way ANOVA, *P<0.05, with *n*=16. (**b**) Analysis of cell numbers in Keap1 KD and Nrf2 OE cells. Quantification of cell numbers was performed 4 and 7 days following plating. Values are given as mean±s.d. Statistical significance was tested with one-way ANOVA, *P<0.05, *n*=3. (**c**). Scratch migration assay of F98 glioma cells with different Nrf2/Keap1 levels. Representative images show that Nrf2 OE and Keap1 KD cells tend to migrate slower than all other groups; scale bar, 200 μm. (**d**) Quantification of the distance of scratch in 0 and 24 hour time points. Values are given as mean±s.d. Statistical significance was tested by two-way ANOVA, *P<0.05, ns: not significant, *n*=6. (**e**) Colony formation assay of F98 glioma cells with different Nrf2/Keap1 levels. Representative images of colonies formed in each group; scale bar, 200 μm. (**f**) Quantification of total colonies area in comparison to controls. Values are given as mean±s.d. Statistical significance was tested by one-way ANOVA, *P<0.05, ns: not significant, *n*=13. (**g**) Extracellular glutamate concentrations measured by high-performance liquid chromatography: Nrf2 OE and Keap1 KD cell groups show higher levels of glutamate in comparison to control cells. Values were given as mean±s.d. Statistical significance was tested by one-way ANOVA, *P<0.05, with *n*=3. ANOVA, analysis of variance.

The different survival rates of glioblastoma patients could be due to various levels of Nrf2 or its functional domains. In order to test this, we investigated the proliferation of Nrf2 and Keap1 challenged cells. First, we performed MTT assays to compare the proliferation rate of cells with various Nrf2 expression levels. The results showed that the Keap1 KD cells as well as Nrf2 OE cells show a significantly higher proliferation rate in relation to controls (Figure 3a). In contrast to this, transfection with the Nrf2 DN vector or siRNA-mediated Nrf2 knockdown (Nrf2 KD) resulted in decreased cell growth to half of the rate found in controls. Moreover, Keap1 OE cells also showed reduced proliferation rates compared to controls (Figure 3a). As an alternative approach, we performed cell count experiments (Figure 3b). As expected, the high proliferation rates of Nrf2 OE and Keap1 KD could be confirmed by these experiments and Nrf2 OE and Keap1 KD gliomas showed almost four to five times higher cell growth in comparison to controls (Figure 3b). Conversely, proliferation of Nrf2 KD, Nrf2 DN and Keap1 OE cells were significantly depressed compared to controls.

Since the glutamate-cysteine antiporter xCT has a pivotal role in the development of brain edema connected to malignant glioma, upstream regulators of xCT are clinically vital candidates for neuroprotective intervention strategies.^16^ Nrf2 is the key regulator and transcription factor of xCT and therefore one could suggest that the poorer survival rate of patients with high Nrf2 expression might be at least in part due to glioma cell growth and a toxic tumor microenvironment. To further examine the contribution of Nrf2 on the morphology and migration of gliomas, we performed cell migration assays. We placed so-called wound scratches on confluent cell layers and monitored the migratory activity of these cells. Surprisingly, the migration analysis revealed that both Nrf2 OE and Keap1 KD cells clearly migrated slower in relation to controls (Figure 3c). The width of the scratch was almost equal in all five groups at the beginning of the experiments. Nevertheless, controls as well as Nrf2 low expressing and dominant negative Nrf2 expressing cells clearly reduced the wound scratch distance after 24 h, while Nrf2 overexpressing cells including Nrf2 OE and Keap1 KD cells migrated slowly. The scratch width measurement after 24 h revealed that cells which express high Nrf2 migrated slower (Figure 3d). These data indicate that elevated Nrf2 levels, either by overexpression of Nrf2 or by knockdown of Keap1 led lower cell migration in comparison to controls. Conversely, decreased Nrf2 led to accelerated migration, indicating that Nrf2 follows the oncogenic concept of ‘To Go or to Grow’.^20, 21^

To further study malignancy, we performed colony forming assay. Representative microscopic images revealed that all high Nrf2 expressing cells (Nrf2 OE and Keap1 KD) have an enhanced ability to form colonies, and cells with reduced Nrf2 expression levels (Nrf2 KD and Keap1 OE) or dominant negative Nrf2 expression (Nrf2 DN) formed smaller and fewer colonies in comparison to the control group. (Figure 3e). Quantification of the total area of colonies in relation to control largely confirmed our observation (Figure 3f). Nrf2 OE and Keap1 KD showed 70 and 50% greater area of colonies in comparison to controls. Knockdown or dominant negative Nrf2 expression led to a 50% decrease in colonies. Interestingly, we did not observe differences between Keap1 OE and controls in this assay (Figure 3f).

**Figure 4:**
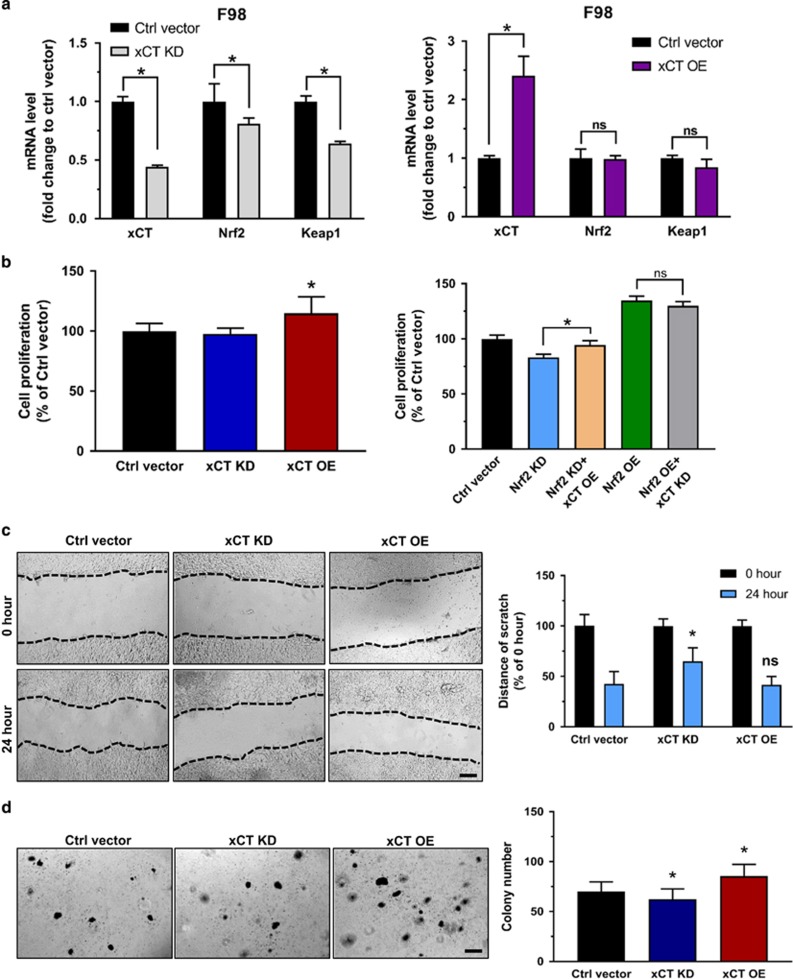
The effect of xCT in glioma cell proliferation, migration and colony formation. (**a**) Quantitive real-time PCR tested the fold change of xCT, Nrf2 and Keap1 mRNA level in F98 glioma cells transfected with xCT KD and xCT OE vectors. Control vector transfected cells served as controls and were normalized to 1. Values are given as mean±s.d. Statistical significance was tested by two-way ANOVA, *P<0.05, *n*=3. (**b**, left) MTT proliferation assay of F98 cell with different xCT expresssion: xCT OE cells showed a slightly higher proliferation rate than control cells (Ctrl vector), while xCT KD cells proliferate as the same rate as control cells. (right) MTT-based proliferation assay of F98 cell with different Nrf2 levels and in addition with transfection of xCT OE or xCT KD vectors. Note that xCT overexpression in Nrf2 KD cells slightly increased the cell proliferation, while xCT KD in Nrf2 OE cells could not significantly depress cell proliferation. Values are given as mean±s.d. Statistical significance was tested by one-way ANOVA, *P<0.05, with *n*=8. (**c**). Scratch migration assay of F98 glioma cells with different xCT levels. (left) Representative images show that xCT KD cells migrate slower than other groups; scale bar, 200 μm. (right) Quantification of the distance of scratch in 0 and 24 hour time points also showed that the scratch of xCT KD cells remained larger in distance compared with control cells, however, xCT OE cells remained the same distance as control. Values are given as mean±s.d. Statistical significance was tested by Two-way ANOVA, *P<0.05, with *n*=10. (**d**) Colony formation assay of F98 glioma cells with different xCT levels. Left, Representative images of colonies forming in each group; scale bar, 200 μm. Right, Quantification of total colonies number in comparison to control cells. Values were given as mean±s.d. Statistical significance was tested by One-way ANOVA, *P<0.05, with *n*=24. ANOVA, analysis of variance.

Next, we studied the impact of Nrf2 on xCT-dependent effects. We speculated that since Nrf2 is a regulator of xCT and thus an expedited Nrf2 expression eventually leads to higher glutamate secretion rates. For this we cultured cells under defined conditions, collected the conditioned medium and performed high-performance liquid chromatography analysis for determining amino acids concentrations in the extracellular space serving as a tumor microenvironment model. We found clear differences in glutamate levels within the Nrf2 OE and Keap1 KD cells. Nrf2 overexpressing cells revealed higher extracellular glutamate levels in comparison to controls. We found the same augmentation in Keap1 KD cells, as Keap1 knockdown led to higher Nrf2 expression (Figure 3g). Thus, these experiments confirm the hypothesis that Nrf2 operates on xCT expression and function leading to a rise of extracellular glutamate. Since xCT is a downstream target of the Nrf2/Keap1 pathway, we next examined the impact of xCT itself on the oncogenic cell activity. First we generated xCT knockdown (xCT KD) F98 glioma cells and xCT overexpressing F98 gliomas (xCT OE). Hence, we tested the corresponding gene expression levels by quantitative real-time PCR analysis. Interestingly, xCT knockdown led to Nrf2 and Keap1 down regulation, whereas xCT overexpression had no effect on Nrf2 and Keap1 mRNA levels (Figure 4a). Moreover, we tested proliferation, migration and colony formation on these cells. Proliferation assay revealed that xCT OE cells grew significantly faster than controls, and xCT KD cells have nearly the same proliferation rate as control cells. In addition, introducing xCT overexpression vectors into Nrf2 KD cells increased cell growth. Knockdown of xCT in the background of Nrf2 OE cells did not significantly inhibit cell proliferation (Figure 4b). However, xCT inhibition in Nrf2 OE cells showed a slower migration rate as well as a decreased capacity of colony formation. In contrast, xCT OE cells showed an increased number of colonies compared to controls (Figures 4c and d). These data indicate that xCT is a valid downstream target of Nrf2 although there might be also other downstream targets or mechanisms involved in Nrf2-mediated oncogenic activities.

### Nrf2 diminishes ferroptotic cell death

**Figure 5:**
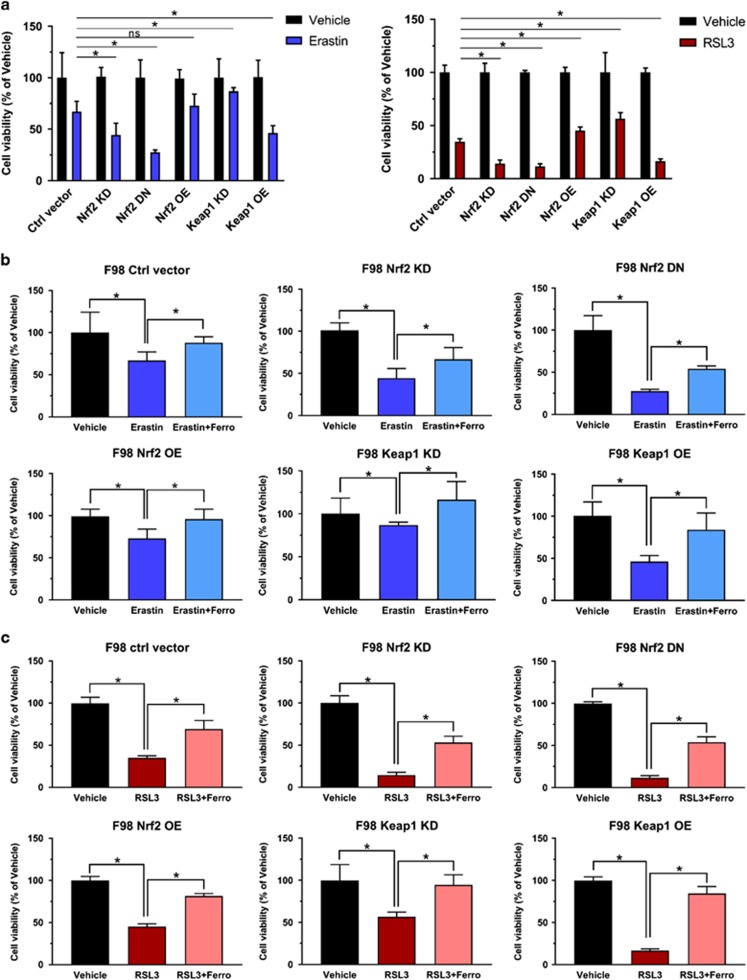
Nrf2-Keap1 pathway induces resistance to ferroptosis in F98 gliomas. (**a**, left) Cell viability analysis following 10 μm erastin treatment in F98 glioma cells expressing different Nrf2/Keap1 vectors. The group with 0.05% (v/v) DMSO treatment (vehicle) served as controls. The O.D. value of vehicle treated cells was set to 100%. Normalized data showed that Nrf2 KD, Nrf2 DN and Keap1 OE glioma cells were significantly more vulnerable to erastin treatment (55%, 70% and 54% decrease respectively vs control 33% decrease in cell viability), while Nrf2 OE and Keap1 KD glioma cells showed more resistant (25% and 13% decrease respectively vs control 33% decrease in cell viability). Values are given as mean±s.d. Statistical significance was tested by One-way ANOVA, *P<0.05, *n*=6. (right) Cell viability under another ferroptosis inducer, RSL3 (0.1 μm), was tested by MTT assay in F98 glioma cells expressing different Nrf2/Keap1 vectors, which showed consistent result as erastin treatment. Values are given as mean±s.d. Statistical significance was tested by One-way ANOVA, *P<0.05, with *n*=6. (**b**) Ferroptosis inhibitor, ferrostatin-1 (Ferro) (1 μm) treatment rescued erastin-induced ferroptosis in each group. Normalized quantification of O.D. value indicated an increase in cell viability of each group respectively: Ctrl vector (21%), Nrf2 KD (20.3%), Nrf2 OE (18.6%), Keap1 KD (34.3%) and Keap1 OE (39%). Values were given as mean±s.d. Statistical significance was tested by one-way ANOVA, *P<0.05, *n*=8. (**c**) 1 μm ferrostatin-1 treatment rescued RSL3-induced ferroptosis in each group. Normalized quantification of O.D. value indicated an increase in cell viability of each group respectively: Ctrl vector (21%), Nrf2 KD (20.3%), Nrf2 OE (18.6%), Keap1 KD (34.3%) and Keap1 OE (39%). Values are given as mean±s.d. Statistical significance was tested by One-way ANOVA, *P<0.05, with *n*=6. ANOVA, analysis of variance.

**Figure 6:**
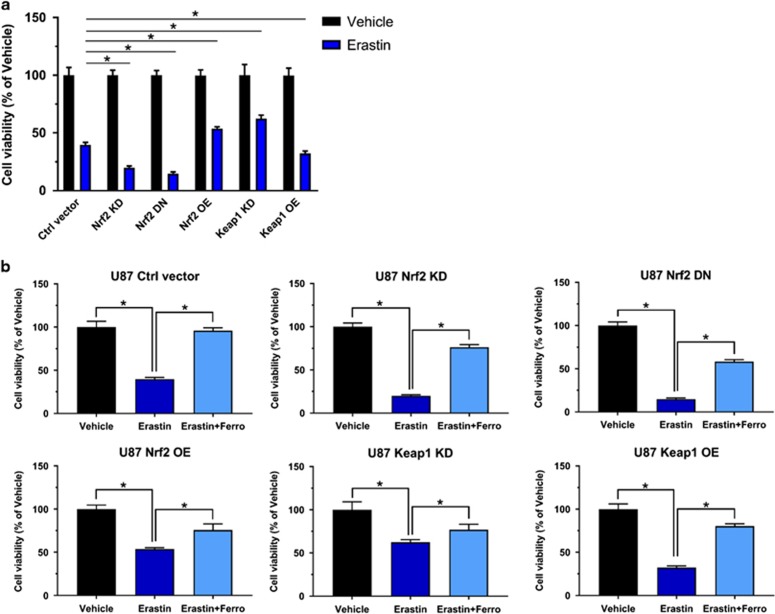
Nrf2-Keap1 pathway induces resistance to ferroptosis in U87 glioma cells. (**a**) Analysis of cell viability under erastin treatment on human glioma cell line U87. DMSO treatment served as vehicle. The O.D. value of vehicle treated cells was set to 100%. Nrf2 KD, Nrf2 DN and Keap1 OE U87 cells showed significantly more vulnerability to 10 μm erastin treatment (80%, 85% and 68% decrease, respectively, vs control’s 60% decrease in cell viability), while Nrf2 OE and Keap1 KD U87 cells were more resistant to erastin (46% and 38% decrease, respectively, vs control’s 60% decrease in cell viability). Values are given as mean±s.d. Statistical significance was tested by One-way ANOVA, *P<0.05, with *n*=6. (**b**) Ferrostatin-1 (1 μm) treatment rescued erastin-induced ferroptosis in each group of U87 cells. Normalized quantification of O.D. value indicated an increase in cell viability of each group respectively: Ctrl vector (56%), Nrf2 KD (56%), Nrf2 OE (43%), Keap1 KD (15%) and Keap1 OE (48%). Values were given as mean±s.d. Statistical significance was tested by one-way ANOVA, *P<0.05, with *n*=6. ANOVA, analysis of variance.

Further we investigated the iron-dependent cell death, termed ferroptosis which has been described as a valid pathway for killing tumor cells.^17, 22^ Erastin and RSL3^23^ are experimentally verified drugs for inducing ferroptosis.^24^ We tested whether Nrf2 and Keap1 expression can challenge the ferroptosis sensitivity of glioma cells. We treated F98 glioma cells expressing different levels of Nrf2 with 10 μm erastin or 0.1 μm RSL3 and 0.05% (v/v) DMSO served as a control. Cell growth assays revealed that Nrf2 OE and Keap1 KD gliomas were less sensitive to erastin and RSL3 compared to control cells. In contrast, Nrf2 KD and Keap1 OE showed increased responsiveness and vulnerability to erastin and RSL3 treatment (Figure 5a). To further verify that erastin or RSL3-induced cell growth inhibition was due to ferroptosis, we treated cells with ferrostatin, a specific ferroptosis inhibitor. These results show that the cell growth was rescued in all groups after ferrostatin treatment (Figure 5b). Interestingly, Nrf2 OE and Keap1 KD cells were almost fully rescued, while control, Nrf2 KD and Keap1 OE cells were solely in part rescued by ferrostatin treatment (Figure 5b). These data also indicates the vulnerability of low Nrf2 expressing gliomas to pharmacological inducers of ferroptosis. Hence, our results were further confirmed in human glioma cells (Figures 6a and b).

### Targeting xCT overcomes Nrf2-Keap1 mediated resistance to ferroptosis

**Figure 7:**
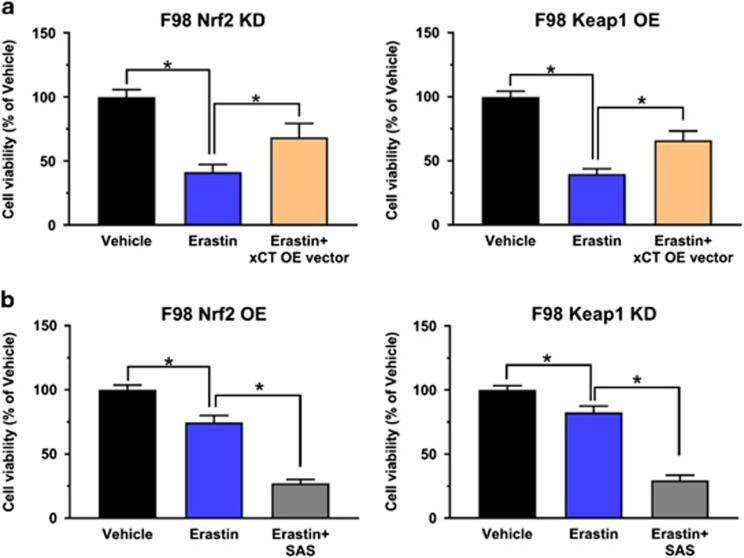
Targeting xCT overcomes Nrf2/Keap1 mediated resistance to ferroptosis. (**a**) xCT overexpression in Nrf2 KD/ Keap1 OE cells rescued their ferroptotic resistance to erastin. Cell viability after 10 μm erastin treatment was tested by MTT assay in F98 glioma cells expressing Nrf2 KD/ Keap1 OE as well as Nrf2 KD plus xCT OE or Keap1 OE plus xCT OE. Normalized data showed that xCT overexpression rescued cell viability by 27% under erastin treatment. Values are given as mean±s.d. Statistical significance was tested by One-way ANOVA, *P<0.05, with *n*=6. (**b**) Pharmacological inhibition of xCT by 400 μm sulfasalazine (SAS) increases the sensitivity of Nrf2 OE/ Keap1 KD cells to erastin treatment. MTT assay showed a 47 and 52% decrease respectively in cell viability when erastin plus SAS treatment was applied. Values are given as mean±s.d. Statistical significance was tested by one-way ANOVA, *P<0.05, with *n*=6. ANOVA, analysis of variance.

Since xCT is a key cellular protection gene regulated by Nrf2 and in addition has also been discovered as a key player in ferroptosis,^25^ we investigated whether Nrf2 mediates its function via xCT expression. Also, we tested whether xCT targeting can overcome Nrf2-Keap1 mediated resistance to ferroptosis.^22^ Interestingly, we found that xCT expression could rescue the effects induced by Nrf2 knockdown (Figure 7a). Conversely, in the background of elevated Nrf2 expression, blocking xCT by pharmacological inhibitor SAS^17^ could dramatically increase their sensitivity to ferroptosis (Figure 7b).

### Knockdown of Nrf2 augments erastin-induced ROS generation in glioma cells

**Figure 8:**
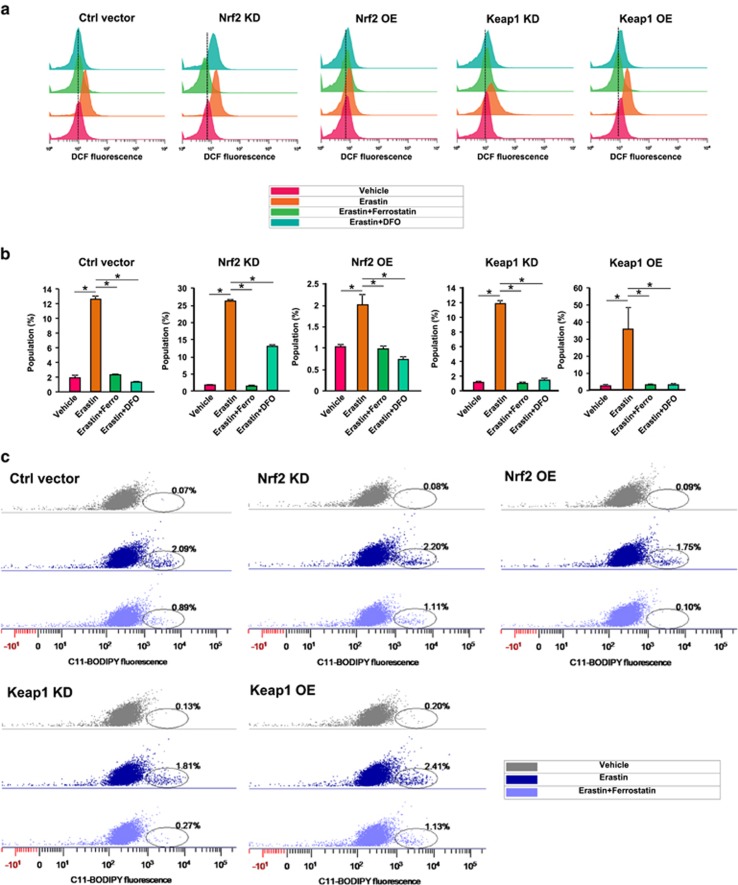
Nrf2-Keap1 regulates erastin-induced ROS generation in glioma cells. (**a**) Fluorescence activated cell sorting-based analysis for total cellular ROS following erastin application in control, Nrf2 KD, Nrf2 OE, Keap1 KD and Keap1 OE glioma cells. Total cellular ROS was monitored via DCF fluorescence intensity. Ferrostatin-1 (1 μm) and DFO (50 μμ) treatment blocked erastin (10 μm) induced ROS generation. Dashed lines indicate the DCF median fluorescence intensity of vehicle treated group. (**b**) Quantification of the percentage of DCF fluorescence positive cell population in various experimental groups. Nrf2 KD and Keap1 OE cells showed 28–30% DCF fluorescence positive cell population after erastin treatment, while Nrf2 OE cells showed only 2% DCF positive population, Keap1 KD and control cells showed a medium 12–13% DCF-positive population. Values are given as mean±s.d. Statistical significance was tested by One-way ANOVA, *P<0.05, with *n*=3. (**c**) Fluorescence activated cell sorting-based analysis for cellular lipid ROS following 10 μm erastin application in control, Nrf2 KD, Nrf2 OE, Keap1 KD and Keap1 OE glioma cells. Lipid ROS was monitored via C11-BODIPY staining. Nrf2 KD and Keap1 OE cells showed higher C11-BODIPY positive population after erastin treatment, while Nrf2 OE and Keap1 KD cells showed a lower C11-BODIPY-positive population compared to controls. Ferrostatin-1 (1 μm) treatment blocked erastin-induced lipid ROS generation. Oval circles indicate C11-BODIPY positive cell population. ANOVA, analysis of variance.

Since erastin-induced ferroptosis is reactive oxygen species (ROS)-dependent,^24^ we measured the total cellular ROS generation after erastin treatment in Nrf2/Keap1 challenged cells using fluorescence activated cell sorting. Noteworthy, in these assays cellular total ROS was further detected by DCF fluorescence after H2DCFDA treatment (Figure 8a). Quantification of fluorescence activated cell sorting analysis revealed that 10 μm erastin treatment led to 25-fold increase of ROS levels in Nrf2 KD cells compared to controls (Figure 8b). In contrast, in Nrf2 OE cells, ROS increased solely two times after erastin treatment (Figure 8b). Further, ROS levels increased 35 times in Keap1 OE cells after erastin treatment which confirms our results found in the Nrf2 KD group. However, Keap1 knockdown did not alleviate erastin-induced ROS generation when compared with controls (Figure 8b). Importantly, all different Nrf2/Keap1 expressing cells showed a massive decrease of ROS level when 1 μm ferrostatin or 50 μm DFO, an iron chelator, was applied (Figure 8b). Based on these data we found that the amount of ROS formation is largely correlated with the vulnerability of glioma cells to erastin treatment. We confirmed this result by measuring lipid ROS formation after erastin treatment (Figure 8b). Compared with control cells, Nrf2 KD and Keap1 OE cells showed a relatively higher lipid ROS formation after erastin treatment, while Nrf2 OE and Keap1 KD cells generated less lipid ROS (Figure 8c). Again, ferrostatin treatment successfully blocked erastin-induced lipid ROS generation (Figure 8c).

### Targeting xCT rebalances lipid ROS generation during glioma ferroptosis

**Figure 9:**
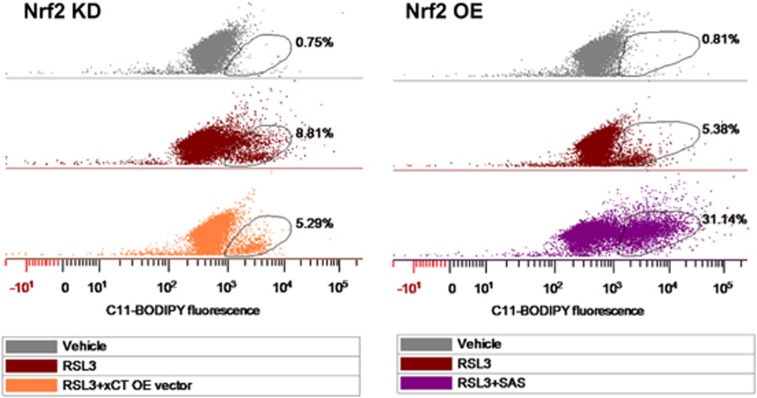
Targeting xCT rebalances lipid ROS generation during glioma ferroptosis. Fluorescence activated cell sorting-based analysis for cellular lipid ROS following 0.1 μm RSL3 application. Lipid ROS was monitored via C11-BODIPY staining. A higher C11-BODIPY positive population was observed in Nrf2 KD cells (8.81%) compared with Nrf2 OE cells (5.38%). Note, Nrf2 KD cells expressing enforced xCT showed decreased C11-BODIPY positive population (5.29%). In addition, when 400 μm sulfasalazine (SAS) applied together with 0.1 μm RSL3 in Nrf2 OE cells, C11-BODIPY positive population dramatically increased (31.14%) compare to the solely RSL3-treated group (5.38%). Oval circles indicate C11-BODIPY-positive cell population.

Next, we tested if targeting xCT can challenge ROS formation during ferroptosis in gliomas with different Nrf2 levels. First we introduced xCT overexpression (xCT OE) into Nrf2 KD cells. Then we measured the amount of lipid ROS formation compared to its counterpart Nrf2 KD cells. After 0.1 μm RSL3 treatment lipid ROS formation was decreased from 8.81% to 5.29% when xCT was overexpressed. Further, since xCT is upregulated in Nrf2 OE cells, we tested whether xCT inhibition in Nrf2 OE cells can reverse lipid ROS formation under RSL3 treatment. As shown, RSL3 treatment alone induced 5.38% lipid ROS-positive population (Figure 9). When RLS3 applied together with the xCT inhibitor SAS at 400 μm, lipid ROS-positive population massively increased to 31.14% compared to controls (Figure 9). These results indicate that xCT is regulated by Nrf2 and has a crucial role in lipid ROS formation during glioma ferroptosis. Conversely, targeting xCT rebalances lipid ROS generation and thereby impacts the sensitivity for glioma cells to ferroptosis.

## Discussion

Here we report on the impact of the Nrf2-Keap1 pathway on oncogenic progression. Northern blot and database analyses showed that Nrf2 is physiological expressed at relatively low levels in various brain regions.^26^ However, in primary brain tumors Nrf2 levels correlate positively with the malignancy of these tumors. Furthermore, increased Nrf2 expression levels are associated with overall poorer survival rates in glioma patients.^26^ The Nrf2 expression level significantly correlated with the prognosis as patients with high expression levels had shorter overall survival.^26^ Thus, malignant gliomas with elevated Nrf2 levels are associated with poor prognosis.^27, 28^

Recent reports showed that Nrf2 knockdown leads to lower proliferation rates *in vitro* and *in vivo* in xenograft experiments such as in melanoma,^29^ cervical cancer,^30^ lung cancer,^31^ gliomas^26^ and pancreatic cancer.^32^ This may be caused by the GSH-induced redox signaling, which is Nrf2-dependent and required for cell cycle progression.^11, 33^ *In vitro* investigations indicated that high levels of Nrf2 correlate with increased proliferation rates. In our study we observed this phenomenom in cells which express high levels of Nrf2 either by Nrf2 overexpression or by breaking the brake of Nrf2 by knocking down Keap1. Interestingly, Keap1 knockdown cells showed higher proliferation rates compared to Nrf2 overexpressing cells. One explanation for this could be that endogenous Keap1 levels are sufficient to bind and degrade heterologous expressed Nrf2 proteins thereby reducing the full Nrf2 transcriptional effects. However, this biological difference could also be due to the fact that Keap1 not only targets Nrf2 for ubiquitination, but also IKKβ, which is an activator of the NFκB-pathway. Thus, depletion of Keap1 can also result in increased activity of NFκB which also contributes to cell proliferation.^34^

Interestingly, Nrf2 does not solely regulates redox homeostasis associated genes but is also able to redirect glucose and glutamine into anabolic pathways.^35, 36^ This is mainly provided by activation of genes involved in the pentose phosphate pathway (for example, glucose-6-phosphate dehydrogenase), the *de novo* nucleotide synthesis (for example, phosphoribosyl pyrophosphate aminotransferase) and the NADPH production (for example, malic enzyme). However, the activation of metabolic genes needs elevated levels of active Nrf2 than what is required for cytoprotection.^36^ Thus, low expression levels of Nrf2 might not be sufficient for a rise of proliferation rate, whereas overexpression of Nrf2 clearly foster cell growth. Furthermore, the colony forming assay revealed that Nrf2 overexpressing cells as well as Nrf2 knockdown cells form increased colony numbers. As the number of colonies in these experiments is a sign for malignancy, this suggests that an alteration of Nrf2 levels in general results in a more malignant status. Nrf2 has been found to affect various cellular pathways. Besides the mentioned effect on proliferation and migration, a high expression of Nrf2 also leads to reduced apoptosis rates^37^ and autophagy.^38^ Furthermore, there are evidences that Nrf2 promotes angiogenesis by activating heme oxygenase 1, which itself takes part in the process of angiogenesis.^39^ In addition, it has already been shown that Nrf2 regulates xCT.^40^ Therefore, it seems likely that at least a part of the effect of Nrf2 on tumor cells is caused by elevated xCT. Thus, such increased Nrf2 expression could contribute to the toxic microenvironment which causes brain edema formation and neuronal degeneration.^16, 41^

In 2008 Shibata *et al.*^42^ showed that loss-of-function mutations in Keap1 lead to a rise in chemo-resistance of gallbladder cancer and furthermore inhibition of Nrf2 leads to enhanced sensitivity to 5-fluorouracile treatment. Hence, therapeutic strategies are generally centered around the contribution of Nrf2 expression to the resistance to radio- and chemotherapy. Still, one should be cautious when it comes to clinical application of Nrf2 inhibitors. Nevertheless, current Nrf2 inhibitory drugs described so far are basically electrophiles scavengers with unspecific activity with broad off-target effects by binding cysteine residues in other proteins and essential enzymes.^43^ Novel screening efforts in a high-throughput manner identified Brusatol which effectively inhibit Nrf2 by degradation. Treatment of A549 lung cancer cells with Brusatol led to enhanced efficiency of a cisplatin therapy.^44^ Such treatment approach was sufficient to reduce GSH levels and sensitized cell lines and xenograft models for chemotherapy. On the other hand, these studies indicate possible disadvantage of increasing Nrf2 protein levels.^43^ This indicats that imbalanced driver gene expression in cancer cannot simply switch on or off, since biological effects often appear in fine tuned thresholds with undefinably or paradox effects (hermesis).^45^

In conclusion, we provide evidence that the Nrf2-Keap1 promotes resistance towards ferroptosis and amplifies oncogenic phenotypes. The operational mode of Nrf2 in gliomas includes xCT augmentation. xCT functions as a malignancy executer for glioma progression and resistance against ferroptotic cell death. Future investigations will concentrate on small molecule inhibition strategies and need to provide an answer whether Nrf2 targeting is specific enough to inhibit cancer growth in glioma patients.

## Material and methods

### Chemicals and reagents

Erastin was purchased from Hycultec GmbH (Beutelsbach, Germany). (1S, 3R)-RSL3 (RSL3) was purchased from Selleckchem (Munich, Germany). Sulfasalazine (SAS) was purchased from Sigma-Aldrich (Taufkirchen, Germany). Erastin, RSL3 and SAS were dissolved in DMSO under sterile conditions to concentration of 100 mm. Desferoxamine (DFO) and ferrostatin-1 (Ferro) were purchased from Sigma-Aldrich. Desferoxamine was dissolved in water under sterile conditions at a concentration of 50 mm. Ferrostatin-1 was prepared in 50% DMSO/water under sterile conditions to a final concentration of 50 mm. Sulfasalazine were dissolved in DMSO under sterile conditions to concentration of 100 mm. Rabbit polyclonal Nrf2 antibody (C-20) and goat polyclonal xCT antibody (Q-18) were purchased from Santa Cruz Biotechnology (Santa Cruz, CA, USA). Mouse monoclonal Keap1 antibody (MAB3024) was purchased from R&D System (Minneapolis, MN, USA). Mouse monoclonal beta-actin antibody (8H10D10) was purchased from cell signaling. H2DCFDA was purchased from Molecular Probes, Invitrogen, (Darmstadt, Germany) and BODIPY C11 (581/591) was purchased from Life Technologies (Darmstadt, Germany). Secondary antibodies used for western blot were obtained from Promega (Madison, WI, USA). Rotifect was obtained from Roth (Karlsruhe, Germany).

### Cell lines and culture condition

Glioma cell lines F98 and U87 were obtained from ATCC/LGC-2397 (Germany) and were cultured under standard condition containing DMEM medium (Biochrom, Berlin, Germany) supplemented with 10% fetale bovine serum (Biochrom), 1% Penicillin/Streptomycin (Biochrom) and 1% Glutamax (Gibco/Invitrogen). Cells were passaged at ~80% confluence by adding trypsin after one PBS wash step and incubated for 5 min, then centrifuged at 900 r.p.m./5 min. Cell lines were transfected according to Broggini *et al.*^46^ Briefly, cells were plated at 20 000/cm^2^ in six-well plates and held under standard conditions. Twenty  hours after seeding, transfection was performed using Roti-Fect (Roth) according to the manufacturer's protocol. Transfected cells were selected with geneticin sulfate 418 and positive cell were expanded (Sigma). For mycoplasma contamination was tested.

### Construction of expression plasmids

The full-length cDNA of Nrf2, Keap1 and xCT (Rat Nrf2 GenBank accession no. NM_031789, Rat Keap1 GenBank accession no. NM_057152 and mouse xCT GenBank accession no. NM_011990) were amplified from different DNA templates using a standard PCR-based cloning strategy. PCR products were cloned and ligated into peGFP-C1 (Takara, Heidelberg, Germany), pmRFP-C1 (BD Clontech, Heidelberg, Germany) and pcDNA3.1 V5-His/lacZ (Invitrogen), according to the manufacture’s instruction respectively. mRNA knockdown vectors were designed according to the critera of Ui-Tei *et al.,*^47^ three 19-mer short interfering RNAs were chosen for RNA interference with rat/human Nrf2 (NM_031789/ NM_006164), Keap1 (NM_057152/NM_203500) and xCT (NM_001107673/NM_01433) transcripts. Cloning of the synthetic oligonucleotids into the pSuperGFP and pSuperRFP vector (Oligoengine, Seattle, WA, USA) were performed by digesting the empty vector with EcoR I and Hind III according to the manufacturer’s instruction. Cells were transfected with different plasmids using Roti-fect according to the manufacture’s instruction. After 5 days, the culture media was refreshed containing 700 μgml^−1^ geneticin sulfate 418 (G418; Sigma) for antibiotic selection. After 3 weeks of culturing in the presence of selection antibiotics, cell clones were further collected. Stably transfected cells were facilitated and maintained under constant 500 μgml^−1^ Geneticin sulfate 418 treatment.

### Database analysis

Analysis of Nrf2 expression in different tissues and cell lines was performed with the Novartis database BioGPS (http://biogps.org) and the CellMiner analysis tool (http://discover.nci.nih.gov/cellminer/home.do). Oncomine (Compendia Biosciences; Ann Arbor, MI, USA; www.oncomine.org) was used to analyze the Nrf2 mRNA expression in different malignant brain tissues. Details of the general standardized normalization techniques and statistical calculations can be found on the Oncomine website (https://www.oncomine.com). Briefly, we perform the normalization per data set and per sample of the microarray data set instead of data set-wide global normalization. Total samples of glioblastoma patients were measured via the U133A microarray. The measured data was processed as described at oncomine.org. For removing differences in dynamic range, a log2 transformation was performed. Kaplan–Meier survival plots for glioblastoma patients with low and intermediate level of Nrf2 mRNA expression were obtained from the Rembrandt database (formerly at https://rembrandt.nci.nih.gov, now moved to http://www.ebi.ac.uk/arrayexpress/experiments/ E-MTAB-3073/ and Georgetown University at https://gdoc.georgetown.edu/gdoc/).

### Cell proliferation analysis

Cell proliferation was measured by using 3-(4,5-dimethylthiazol-2-yl)-2,5-diphenyl-tetrazolium-bromide (MTT) assay or cell counter. Cells were plated at 3000 cells/cm^2^ in 96-well plate and incubated at standard conditions for certain days. At measure point cells were incubated with MTT solution (Roth) (5 mgml^−1^) at 37 °C, 5%CO_2_ for 4 h. After 4 h incubation the MTT solution was removed and cells were lysed with 100 μl Isopropanol+ 0.1N HCl and OD value was measured with SLT spectra (Crailsheim, Germany) using Tecan X Fluor4 software (Männedorf, Switzerland). For cell counting experiment, cells were washed once with PBS, then trypsinized with 200 μl and incubated for 5 min and reaction was stopped with 800 μl culture medium. After thoroughly suspension samples were counted using cell counter Z2 (Beckmann-Coulter, Brea, CA, USA) using 100 μl sample and 9.9 ml isotonic 2 solution.

### RNA isolation and quantitative RT-PCR experiments

For each cell line one cell culture flask with 60–80% confluent cells was used for RNA isolation. Cells were washed with PBS once, trypsinized for 5 min and centrifuged for 5 min at 900 r.p.m. The cell pellet was dissolved in 200 μl PBS and RNA was isolated using the High Pure RNA Isolation Kit (Roche, Basel, Switzerland) according to the user manual. RNA concentration was quantified by NanoVue Plus Spectrophotometer (GE Healthcare, Solingen, Germany). cDNA synthesis was performed with SuperScript III Reverse Transcriptase (Invitrogen). Quantitative real-time PCR was performed with SYBR Green PCR master mix (Qiagen, Hilden, Germany). The oligos used in this study are: Rat/Human Nrf2 forward primer: 5′-TCTGACTCCGGCATTTCACT-3′; Rat/Human Nrf2 reverse primer: 5′-GGCACTGTCTAGCTCTTCCA-3′. Rat xCT forward primer: 5′-1TGCTGGCTTTTGTTCGAGTCT-3′ Rat xCT reverse primer: 5′-GCAGTAGCTCCAGGGCGTA-3′. Human xCT forward primer: 5'-CCCAGATATGCATCGTCCTT-3′; Human xCT reverse primer: 5′-GCAACCATGAAGAGGCATGT-3′ Rat/Human Keap1 forward primer: 5′-TTCGCCTACACGGCCTC-3′ Rat/Human Keap1 reverse primer: 5′-GAAGTTGGCGATGCCGATG-3′; Rat/Human GAPDH forward primer: 5′-TGCACCACCAACTGCTTAGC-3′; Rat/ Human GAPDH reverse primer: 5'-GGCATGGACTGTGGTCATGA-3′. Rat/Human beta-actin forward primer: 5′-GCTCCTCCTGAGCGCAAG-3′; Rat/Human beta-actin reverse primer: 5'-CATCTGCTGGAAGGTGGACA-3′. Real-time cycling parameters: initial activation step (95 °C, 15 min), cycling step (denaturation 94 °C, 15 s; annealing at 60 °C, 30 s; and finally extension for 72 °C, 30 s x40 cycles), followed by a melting curve analysis to confirm specificity of the PCR. The Ct value was corrected by Ct reading of corresponding GAPDH or Beta-actin controls. The reaction was performed using Light Cycler 480 (Roche).

### Immunoblotting

80% confluent cells were directly lysed in 6-well plate with NP 40 buffer containing 5 mm NaF and a protease inhibitor cocktail (Roche) and homogenized by ultrasound (Bandelin Sonoplus, Berlin, Germany). In brief, following 20 min incubation on ice, samples were centrifuged at 8000 r.p.m. for 10 min. Supernatants were isolated and protein concentration measured with the NanoVue Plus Spectrophotometer (GE Healthcare). Samples were mixed with loading buffer (4 ×) and NuPAGE Sample Reducing Agent (10 ×) (Invitrogen) and boiled at 96 °C for 8 min. Equal amounts of protein lysates were loaded and separated by 10–12% SDS-NuPage gel (Invitrogen) and electrophoresis was performed in MOPS-buffer, transferred to polyvinylidene difluoride membranes (Roth). Pretreatment of membranes was performed in PBS containing 2% Magic block (containing 10% Top block (Lubio Science, Luzern, Switzerland)) for 1 h. The membranes were then hybridized with antibodies against Nrf2 (1:200), Keap1 (1:1000), xCT (1:200) and β-actin (1:3000) in 5% BSA-TBST for 10 h at 4 °C. ECL detection was conducted with horseradish peroxidase-conjugated secondary antibody incubation and ECL plus kit (GE Healthcare).

### Migration assay

Cell migration assays were carried out as previously described.^48^ In brief, cells were plated in 24 wells at 15 000 cells/cm^2^ and held under standard conditions until confluence of 80%. Wound scratch was set using a 200 μl pipette. Floating cells were carefully removed. Plates were then held under standard conditions for additional 24 h. Pictures were taken with an Olympus IX71 microscope (Tokyo, Japan) at different time points and analyzed with Image J software (NIH, Bethesda, MD, USA) by measuring distance between the migrating cell boundaries.

### Colony forming assay

The colony forming assay was adapted with modifications from Zhang *et al.*^49^ Briefly, cells were seeded (2000/cm^2^) in 1% soft agar on top of a 2% soft agar layer. Cells were cultured under standard condition for 10 days. Four pictures per well were taken with Olympus x71 and numbers of colonies were counted manually. The colony number and area were analyzed with Image J software (NIH).

### Amino acid profiling of glioma conditioned medium

Cells were seeded in six-well plates at a density of 75 000 cells per well in full DMEM medium. For the experiments at least three wells per cell line were used. After incubation overnight, the medium was changed to only DMEM without any supplements. After incubating for another 48 h, medium was collected and measurement was performed by using high-performance liquid chromatography. Amino acids were analyzed by ion-exchange chromatography and post-column ninhydrin derivatization technique using a fully automated amino acids analyzer (Biochrom 30+, Laborservice Onken). For the amino acid analysis, 100 μl of sample was deproteinised with 100 μl of 10% sulphosalicylic acids. 20 μl of this supernatant was then loaded by the autosampler into a cation-exchange resin-filled column.

### Fluorescence activated cell sorting-based analysis

80 000 cells/well were seeded in six-well plates and treated with 0.05% DMSO (control),10 μm erastin/0.1 μm RSL3, 10 μm erastin/0.1 μm RSL3 plus 1 μm ferrostatin-1 and 10 μm erastin plus 50 μm DFO for 8 h. Cells were collected by centrifuge, the pellets were washed with PBS and afterwards re-suspend in 10 μm H_2_DCFDA or 2 μm C11-BODIPY diluted in PBS. ROS analysis was performed within 1 h after H2DCFDA or C11-BODIPY treatment with Flow Cytometer BD FACSCanto II (BD Bioscience, Heidelberg, Germany). Analyses were carried out with Flowing Software 2 (Turku Center for Biotecnology, University Turku, Turku, Finland), and Winlist FACS software (Verity Software House, Topsham, ME, USA).

### Statistical analysis

Statistical analysis was performed by GraphPad Prism 7 (GraphPad Software, Inc., La Jolla, CA, USA). Data are expressed as mean±s.d. taken from at least three independent experiments if not otherwise indicated. Means were compared with one-way analysis of variance or two-way analysis of variance (when comparing more than two groups). Multiple comparisons was performed by Tukey’s multiple comparisons test. *P*-values of *P<0.05 was considered as statistically significant. Estimated effect size and observed power were analyzed by IBM SPSS Statistics 23.0 software (IBM, Armonk, NY, USA). Sample size was adjusted to reach statistical power of 80% or more. Exclusion criteria was pre-established, values out of measuring range, and samples that failed of treatment were excluded.
